# Protocol and methods for testing the efficacy of well-being therapy in chronic migraine patients: a randomized controlled trial

**DOI:** 10.1186/s13063-018-2944-5

**Published:** 2018-10-16

**Authors:** Giovanni Mansueto, Francesco De Cesaris, Pierangelo Geppetti, Fiammetta Cosci

**Affiliations:** 1 Department of Health Sciences, University of Florence, via di San Salvi 12, 50135 Florence, Italy; 2 Department of Psychiatry & Neuropsychology, Maastricht University, Maastricht, The Netherlands; 3 Headache and Clinical Pharmacology Center, Azienda Ospedaliero-Universitaria Careggi, Florence, Italy

**Keywords:** chronic migraine, migraine, headache, well-being therapy, psychological well-being, psychotherapy

## Abstract

**Background:**

Chronic migraine is a chronic medical condition associated with resistance to pharmacological treatment and poor benefits from the psychological interventions studied to date, including acceptance and commitment therapy or mindfulness. This manuscript describes the rationale and methods for a pilot feasibility study designed to (1) establish and (2) evaluate the feasibility and acceptability of research procedures and interventions to investigate whether well-being therapy improves outcomes relative to a control condition.

**Methods:**

The current intervention will use a randomized controlled trial design, wherein 30 outpatients with chronic migraine will be randomized (1:1) to well-being therapy (*n* = 15) or to a control condition (*n* = 15). Primary outcomes include the level of disability caused by migraine and the frequency, duration, and intensity of migraine attacks; the secondary outcomes focus on anxiety, depression, psychological well-being, euthymia, and distress. Primary and secondary outcomes will be assessed at baseline, after sessions 4 and 8, and at 3-month follow-up. The Ethical Review Boards at the University-Hospital Careggi has approved the study (5th December 2017).

**Discussion:**

Identifying medium-term interventions able to improve chronic migraine is relevant to manage this illness. The present randomized trial might represent a step forward for managing chronic migraine by means of psychological interventions.

**Trial registration:**

ClinicalTrial.gov Identifier: NCT03404336. Registered on 19 January 2018.

**Electronic supplementary material:**

The online version of this article (10.1186/s13063-018-2944-5) contains supplementary material, which is available to authorized users.

## Background

Migraine is a prevalent disabling condition affecting approximately 15% of subjects in the general population [1]. Migraine can be episodic (i.e., less than 15 headache days per month) or chronic (15 or more headache days per month for at least 3 months) [2–5], the latter affecting 1–3% of the general population [6]. Chronic migraine is the most disabling form of migraine, resulting in lower socioeconomic status and health-related quality of life as well as increased headache-related burden [6]. Chronic migraine is often resistant to treatment [7] and the efficacy of stress-oriented psychotherapeutic interventions, such as acceptance and commitment therapy and mindfulness, has been shown to be poor [8–18].

Well-being therapy (WBT) is a short-term (i.e., 8 sessions) well-being-oriented psychotherapeutic strategy emphasizing self-observation of patient’s well-being with the use of a structured diary, interaction between patients and therapists, and homework [19–21]. It is based on the model of psychological well-being developed by Jahoda in 1958 [22] and further refined by Ryff [23]. The goals of WBT are the improvement of the psychological well-being and the achievement of a state of euthymia [19–21]. In previous randomized controlled trials, WBT was found to be efficacious in reducing relapse rates in depressed adults [24, 25] and in treating generalized anxiety disorder [26] and cyclothymia [27], suggesting that psychological well-being may be increased by a specific psychotherapeutic method and that such increase may yield a protective and preventive effect. WBT was also shown to be efficacious when distress-oriented psychotherapeutic interventions did not produce benefits [28].

We designed a pilot study to (1) establish and (2) evaluate the feasibility and acceptability of research procedures and interventions to investigate whether WBT improves outcomes relative to a control condition in chronic migraine patients who were resistant to pharmacological treatment. Although WBT might be a complementary therapeutic option [29], no studies have addressed the potential of such psychological intervention for chronic migraine. The present manuscript describes the study design and procedures for a pilot randomized controlled trial investigating feasibility, acceptability, and effects of WBT in chronic migraine patients.

## Methods

This protocol is reported in accordance to the Standard Protocol Items: Recommendation for Intervention (SPIRIT) guidelines [30, 31]. For the SPIRIT Checklist, see Additional file 1.

### Study overview

**Fig. 1:**
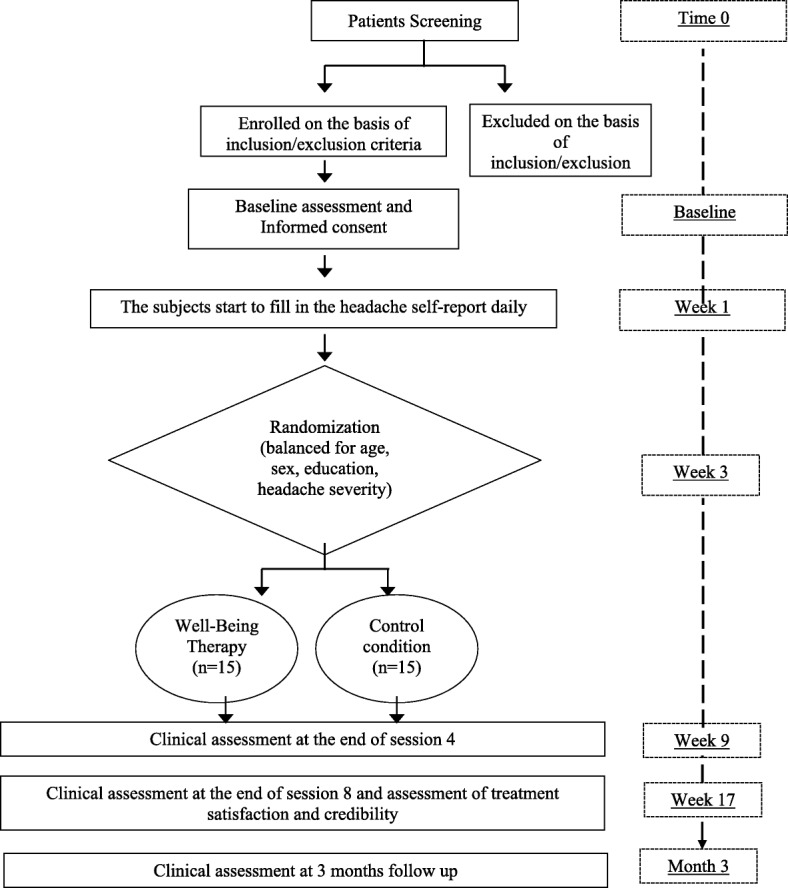
Summary of study timeline. *PT* post treatment assessment, *3 M* 3-month follow-up

This is a 16-week, stage 1b, single site study designed to evaluate the feasibility, acceptability, and preliminary effects of two psychological interventions in chronic migraine patients. It was designed to evaluate the feasibility of conducting a two-arm pilot randomized controlled trial with participants randomized (1:1) to WBT or to a control condition. Primary and secondary outcomes will be assessed at baseline, after sessions 4 and 8, and at 3-month follow-up. Figure 1 provides an overview of the study design and timeline.

### Specific aims

The specific aims and hypotheses for this pilot trial are (1) to examine, from baseline to 3 month follow-up, the level of disability due to migraine and the frequency, duration, and intensity of migraine attacks. We hypothesize that patients receiving WBT will show a better outcome compared to those assigned to the control condition (primary outcome); and (2) to examine, from baseline to 3 month follow-up, the level of anxiety, depression, psychological well-being, euthymia, and distress. We hypothesize that patients receiving WBT will show lower anxiety, depression, and distress, and greater psychological well-being compared to those assigned to the control condition (secondary outcome).

### Participant selection recruitment and retention

Eligibility criteria for this ongoing pilot trial (target *n* = 30) include (1) 18–65 years of age; (2) Italian mother tongue; (3) a diagnosis of chronic migraine according to the International Classification of Headache Disorders [2], thus presenting specific features (i.e., unilateral and pulsating pain of moderate or severe intensity, which is aggravated or precipitated by routine physical activities and is combined with nausea and/or vomiting, photophobia, and phonophobia) and migraine headache on 15 or more days per month; (4) headache chronicity for a minimum of 1 year and pattern of headache symptoms stable for a period of at least 6 months [32]; (5) no pharmacological therapy or dietary supplement use for chronic migraine or pharmacological therapy/dietary supplement use for chronic migraine stable for at least 3 months; and (6) psychotropic medication allowed only if stable for at least 3 months.

Exclusion criteria are (1) a diagnosis of headaches due to medication overuse; (2) co-occurrence of psychiatric disorder(s) according to the Diagnostic and Statistical Manual of mental disorders 5th edition (DSM-5) [33], as diagnosed via the MINI International Neuropsychiatric Interview [34]; (3) co-occurrence of chronic unstable medical conditions [32]; (4) being pregnant or lactating [32]; (5) under exogeneous hormone treatment (i.e., hormonal contraceptives, postmenopausal hormone therapy) [32]; or (6) any other condition that, according to the investigators’ opinion, may alter the ability of the patient to follow study procedures.

Participants will be recruited from the SOD Headache Centre and Pharmacologic Clinic of the Careggi University Hospital of Florence. Research assistants (RAs) will screen participants for preliminary eligibility and those who meet initial eligibility criteria will be asked to attend the clinic for an in-person evaluation where informed consent is obtained. The current study employs several measures to minimize attrition. All participants will be assessed by clinical psychologists who will collect sociodemographic and clinical data.

### Randomization

Following eligibility determination, participants will be randomly assigned to either WBT or a control condition. Block randomization of size two will be used. The two groups will be balanced for age, sex, education, and headache severity. The allocation schedule has been created by the principal investigator (FC) using a computerized random number generator and is concealed to the investigators responsible for enrollment. When a registered patient is randomized, the investigator will contact the principal investigator who will communicate the assigned treatment group. Single blindness will be ensured.

#### Study interventions

**Fig. 2:**
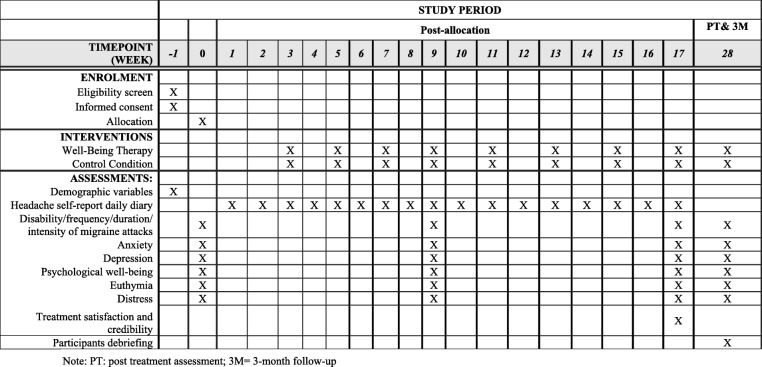
Two-arm randomized controlled trial design and data collection schedule

Figure 2 provides an overview of the study procedure.

### Well-being therapy (WBT)

WBT is used as the only non-pharmacological therapeutic strategy. Eight sessions (Table 1) will be delivered every other week, with a duration of 60 min each. The manualized WBT will be used [19–21]. The initial phase is concerned with self-observation of psychological well-being. Once the instance of well-being is properly recognized, the patient is encouraged to identify thoughts, beliefs, and behaviors leading to premature interruption of well-being (intermediate phase). The final part involves cognitive restructuring of dysfunctional dimensions of psychological well-being and meeting the challenge that optimal experiences may entail [19–21]. An assessment of treatment adherence, satisfaction, and side effects will be performed via ad hoc questions.

**Table 1:** Well-being therapy (WBT) sessions according to Fava [19, 21] and Fava et al. [20]

Session	Focus	Objectives	Tool
Session 1	Identifying and setting episodes of well-being into situational context	Report the circumstances surrounding the episodes of well-being rated on a scale of 0 to 100, with zero being absence of well-being and 100 being the most intense well-being	Diary
Session 2	Optimal experiences	Define optimal experiences and report on them	Diary
Session 3	Identifying interfering thoughts and behaviors	Report the thoughts and behaviors that interrupt well-being	Diary
Session 4	Illustrating autonomy	Proposing the dimension of autonomy as material for reflections to the well-being diary	Examples, metaphors
	Reflecting and practicing autonomy	Attempting to explain the premature interruption of well-being with the help of the dimension of autonomy	Daily exposure to pleasurable activities and diary
Session 5	Illustrating environmental mastery	Proposing the dimension of environmental mastery as material for reflections to the well-being diary	Examples, metaphors
	Reflecting and practicing environmental mastery	Attempting to explain the premature interruption of well-being with the help of the dimension of environmental mastery	Daily exposure to pleasurable activities and diary
Session 6	Illustrating positive relations with others	Proposing the dimension of satisfactory interactions as material for reflections to the well-being diary	Examples, metaphors
	Reflecting and practicing positive relations with others	Attempting to explain the premature interruption of well-being with the help of the dimension of satisfactory interactions	Daily exposure to pleasurable activities and diary
	Illustrating personal growth	Proposing the dimension of individual’s style and degree of growth as material for reflections to the well-being diary	Examples, metaphors
	Reflecting and practicing personal growth	Attempting to explain the premature interruption of well-being with the help of the dimension of individual’s style and degree of growth	Daily exposure to pleasurable activities + diary
Session 7	Illustrating self-acceptance	Proposing the dimension of development or self-actualization as material for reflections to the well-being diary	Examples, metaphors
	Reflecting and practicing self-acceptance	Attempting to explain the premature interruption of well-being with the help of the dimension of development or self-actualization	Daily exposure to pleasurable activities and diary
	Illustrating purpose in life	Proposing the dimension of an individual’s balance and integration of psychic forces as material for reflections to the well-being diary	Examples, metaphors
	Reflecting and practicing purpose in life	Attempting to explain the premature interruption of well-being with the help of the dimension of an individual’s balance and integration of psychic forces	Daily exposure to pleasurable activities and diary
Session 8	Placing the experience of WBT in the treatment history of the patient Ending treatment therapy	Review the patient’s effort to contrast interruptions of well-being Continuing cognitive restructuring and in vivo contrast of automatic thoughts Checking the patient’s feeling about ending the therapy	Diary

### Control condition

The control condition will include 8 bi-weekly sessions based on the Lifestyle and Well-being National Institute for Health and Care Excellence (NICE) guidelines (https://www.nice.org.uk/guidance/lifestyle-and-wellbeing) and on the World Health Organization 12 Steps to Healthy Eating (http://www.euro.who.int/en/health-topics/disease-prevention/nutrition/a-healthy-lifestyle). These sessions (Table 2) will inform participants about well-being and the lifestyles that can influence it. No access to specific WBT ingredients will be allowed. An assessment of treatment adherence, satisfaction, and side effects will be performed via ad hoc questions.

**Table 2:** Control condition according to the NICE guidelines [34] and World Health Organization-12 Steps to Healthy Eating (http://www.euro.who.int/en/health-topics/disease-prevention/nutrition/a-healthy-lifestyle)

Sessions	Focus
Session 1	Illustrating the concept of lifestyle and well-being
Session 2	Illustrating healthy eating and steps to healthy eating
Session 3	
Session 4	Illustrating physical exercise and how it promotes health
Session 5	Illustrating smoking and tobacco and how they can damage health
Session 6	Illustrating alcohol and how it can damage health
Session 7	Illustrating drug misuse and how it can damage health
Session 8	Illustrating sexual health

#### Monitoring

The subjects will fill in the headache self-report daily diary from 2 weeks before the first session of therapy. Thereafter, they will receive the WBT or the control intervention. The subjects will be re-assessed at the end of session 4 of treatment, at the end of session 8 of treatment, and at 3-month follow-up. Since participants will not be informed if they receive WBT or the control condition, an assessment of treatment credibility will be provided at the end of session 8. At the conclusion of the study, participants will be debriefed and informed that the control condition was necessary to test the study hypothesis.

#### Assessments

Measures selected in this study mirror those commonly used in treatment outcome trials for chronic migraine [9, 13, 15, 16] and are consistent with the international expert consensus guidelines for determining treatment response [35, 36]. These measures include those for screening and eligibility, assessing feasibility, acceptability and patient satisfaction, monitoring patient safety, and determining primary and secondary clinical outcomes. Unless otherwise specified, all measures, including primary and secondary outcome measures, will be administered and collected by Ph.D. level independent evaluators (IEs) naive to study condition.

### Eligibility determination and diagnostic assessment

The diagnosis of chronic migraine will be assessed for the determination of eligibility by the clinical interview according to the International Classification of Headache Disorders [2]. Information on psychological and pharmacological treatment history and co-occurrence of chronic unstable medical conditions will be collected via ad hoc questions previously used [37]. The co-occurrence of psychiatric disorders will be assessed via the MINI International Neuropsychiatric Interview [34], a short structured diagnostic interview validated against the Structured Clinical Interview for DSM diagnoses and against the Composite International Diagnostic Interview for ICD diagnoses as well as against expert opinion in a large sample in four European countries (France, United Kingdom, Italy, and Spain). We will use the Italian version 7.0.0, which allows diagnosis formulation according to the DSM-5 [33].

### Migraine and headache assessment

The level of pain and disability caused by migraines in the patient’s life will be measured using the Migraine Disability Assessment Score questionnaire [38, 39], a 5-item self-administered questionnaire evaluating the influence of migraine on three domains of activity, namely paid work or school, household work, and family, social or leisure activities, over the preceding 3 months; higher scores indicate more severe disability. The Migraine Disability Assessment Score questionnaire showed good internal consistency and high reliability [39]. The level of headache experienced will be assessed via a daily self-report headache diary built according to the guidelines provided by Penzien et al. [32] for trials of behavioral treatments for headache and according to the guidelines provided by Tfelt-Hansen et al. [36] for controlled trials of drugs in migraine treatment. The diary collects information on headache frequency, average headache severity (0 = no headache, 1 = mild headache, 2 = moderate headache, 3 = severe headache), duration of peak headache in hours (start time, end time), intake of symptomatic headache treatments (dose), headache relief after 2 h (0 = no headache, 1 = mild headache, 2 = moderate headache, 3 = severe headache), functional disability for the day scale (0 = no disability: able to function normally; 1 = performance of daily activities mildly impaired: can still do everything but with difficulties; 2 = performance of daily activities moderately impaired: unable to do some things; 3 = performance of daily activities severely impaired: cannot do all or most things, bed rest may be necessary).

### Feasibility, acceptability, and participant satisfaction

Measures of feasibility include the ability to recruit the intended population from the SOD Headache Centre and Pharmacologic Clinic of the University Hospital Careggi of Florence, participant willingness to be randomized, and session attendance. In addition, the widely used Client Satisfaction Questionnaire-8 [40] is proposed. The credibility of treatment rationale and expectancy for improvement are evaluated via the Credibility Expectancy Questionnaire [41], which has been shown to be predictive of clinical outcomes in previous treatment trials and was adapted to include questions on expectancies regarding the likelihood of reduction of headache symptoms.

To maintain the blindness of the IEs, these acceptability and satisfaction measures will be administered at the end of the treatment period (session 8) by RAs not otherwise involved in the study.

### Primary outcomes

The level of disability caused by migraine and the frequency, duration, and intensity of migraine attacks are the primary clinical outcomes, measured at baseline (prior to randomization), at the end of sessions 4 and 8 of the treatment, and at 3-month follow-up.

### Secondary outcomes

Secondary outcomes focus on anxiety, depression, psychological well-being, euthymia, and distress. These variables are assessed at baseline (prior to randomization), at the end of session 4 and 8 of the treatment, and at 3-month follow-up. Anxiety and depression will be assessed via the Symptom Questionnaire [42, 43], a self-administered 92-item dichotomous scale (yes/no or true/false), of which 68 items indicate symptoms (symptom subscales) and 24 items are antonyms of some of the symptoms and indicate well-being (well-being subscale). Higher subscale scores indicate higher severity of symptoms or higher well-being, respectively. The Symptom Questionnaire had good validity and reliability [43]. Psychological well-being will be assessed via (1) the World Health Organization-Five Well-Being Index [44], a self-administered 5-item scale, with answers rated on a 6-point Likert scale assessing well-being in the 2 previous weeks, wherein the higher the score, the higher the level of well-being; and (2) the Psychological Well-Being Questionnaire [45], a 84-item self-administered inventory, rated on a 6-point Likert scale, measuring six constructs, namely Autonomy (i.e., independence and self-determination); Environmental Mastery (i.e., ability to manage one’s life); Personal Growth (i.e., being open to new experiences); Positive Relations with others (i.e., having satisfying relationships); Purpose in Life (i.e., believing that one’s life is meaningful); and Self-Acceptance (i.e., having a positive attitude towards oneself and past life). The Psychological Well-Being Questionnaire has shown good psychometric properties [46].

Euthymia will be assessed via the Euthymia scale [47], a self-administered scale consisting of 10 items with a dichotomous response mode (yes/no or true/false). The higher the score, the higher the level of euthymia [47]. Distress will be measured via the Psychosocial Index [48], a 55-item questionnaire assessing stress, wellness, illness behavior, psychological distress, and quality of life; it showed good psychometric properties [49]. Some questions of the Psychosocial Index require specific responses, most of them provide a dichotomous response, others are measured on a 4-point Likert scale. The final item, on the quality of life, has five possible answers.

### Assessment training

All IEs are Ph.D.-level researchers with extensive prior diagnostic assessment experience. Regardless of familiarity with study assessment measures, all IEs will receive additional training on the primary and secondary clinical outcome measures prior to conducting study assessments. This includes training on viewing and rating clinical outcome measures, created by the principal investigator (FC). RAs will follow our standard training protocol under the direction of the study investigators. This structured training protocol consists of a graduated set of tasks and experiences, beginning with reading relevant papers, studying instruments and instruction booklets, multi-day didactics, and reviewing suggestions for handling common interviewing problems. RAs are closely supervised during these training sessions and during their initial interviews by the principal investigator (FC).

### Data management and statistical analysis plan

#### Data collection, management and assurance of quality

Data from the self-report questionnaires, self-report headache diary, and the MINI will be first recorded on paper forms and then entered into the SPSS 20.0 (Statistical Package for Social Science) [50] macro and verified by research staff. Several data monitoring procedures will be implemented to ensure data quality, including (1) systematically recording study notes-to-file in the case of any event that threatens data integrity and (2) routine internal audits confirming proper informed consent, accurate completion of data forms, and documentation of missing data.

#### Data analysis plan

We will primarily focus on descriptive statistics to examine both feasibility, acceptability, and outcomes. This includes examining rates of study recruitment, participant’s willingness to be randomized, session attendance, and patient satisfaction. Additionally, we will compare rates of migraine attacks per month and their duration as well as the level of disability due to migraine and psychological measures. We will also examine the means of key variables (i.e., level of pain and disability caused by migraine, frequency, duration, and intensity of migraine attacks, anxiety, depression, psychological well-being, euthymia, distress) across baseline, session 4, session 8, and 3-month follow-up. We will use ANCOVA to compare means of variables [51], χ^2^ to compare frequencies, and a multivariate logistic regression analyses to identify independent predictors of psychological well-being among socio-demographic and psychological variables measured at baseline. The number of migraine attacks are recorded via the headache self-report daily diary following the rules suggested by Tfelt-Hansen et al. [36], namely (1) a migraine attack which is interrupted by sleep, or temporarily remits, and then recurs within 48 h is recorded as one attack; (2) an attack treated successfully with medication but with relapse within 48 h counts as one attack; and (3) a practical solution to differing these using diary entries over the previous month is to count as distinct attacks only those that are separated by an entire day headache free.

#### Power calculation

The sample calculation was run via an a priori sample size calculator for Student’s *t* test. Cohen’s d was calculated on the basis of the results of (1) Mo’tamedi et al. [14], concerning the Migraine Disability Assessment scale score under acceptance and commitment therapy and under the control condition (this is the only study conducted in chronic migraine patients in which the Migraine Disability Assessment scale [38], which is one of our primary outcome measures, was used, although it tests the efficacy of a stress-oriented psychotherapeutic intervention which is different from WBT) and (2) Fava et al. [26], which tests the efficacy of WBT in a sample of patients with generalized anxiety disorder using, as the outcome measure, the Psychological Well-being scale [49], which is one of our secondary outcome measures. A Cohen’s d of 2.76 was obtained from Mo’tamedi et al. [14] and a Cohen’s d of 1.38 was obtained from Fava et al. [26]. Setting the power at 0.95 and the two-sided alpha at 0.05, a minimum sample size of 5 per group (two-tailed hypothesis) was obtained on the basis of Mo’tamedi et al. [14] and a minimum sample size of 15 per group (two-tailed hypothesis) was obtained on the basis of Fava et al. [26]. Thus, we will enroll 15 subjects per group for a total of 30 subjects. In case of drop-out, new enrolment will be run in order to have a final sample size of 15 subjects per group who complete the trial.

#### Ethics issues

No invasive procedure/test will be performed and no drug administrated. During the study (from the signature of informed consent to the study termination), any clinical event will be registered in a case report form. There are no documented side effects due to WBT [19–21] or the control condition.

## Discussion

The pharmacological treatment of chronic migraine has achieved remarkable progress in recent years [52, 53]. However, significant issues persist in patients who do not benefit from current drug treatments [54, 55] or for those with limited access to innovative approaches, such as anti-calcitonin gene-related peptide monoclonal antibodies, due to financial constraints. Current evidence on the efficacy of psychological interventions remains limited and unsatisfactory [8–18]. This study proposes a method aimed to empirically evaluate whether WBT provides a beneficial effect in chronic migraine patients. In particular, the present pilot study aims to test WBT versus a control condition as a psychological intervention for chronic migraine. The randomized trial represents a step towards the management of chronic migraine by means of psychological interventions.

We acknowledge some limitations of the study. Inclusion and exclusion criteria are proposed to maximize the safety of enrolled patients. However, such restrictions may reduce the possibility of generalization of results to the patient population with a more severe condition or those with high degrees of comorbidity. In addition, the sample size will not allow to test for potential differences in outcome between different durations of the chronic migraine or diverse levels of severity. Further, several key questions remain and could be the object for future studies, including whether, and at what size, differences in patient motivation or in the clinician–patient relationship may affect the outcomes. Should results of this pilot exploratory trial support our hypothesis, the next step will be a second trial enrolling a larger number of chronic migraine patients with a randomized and controlled multicentric design to investigate efficacy and potential mediators of treatment effects.

### Trial status

Participant recruitment has not started.

## Additional file

Additional file 1: SPIRIT 2013 Checklist: Recommended items to address in a clinical trial protocol and related documents. (DOC 122 kb)

## Abbreviations

DSM: Diagnostic and Statistical Manual of mental disorders
IEs: independent evaluators
RAs: research assistants
WBT: well-being therapy
